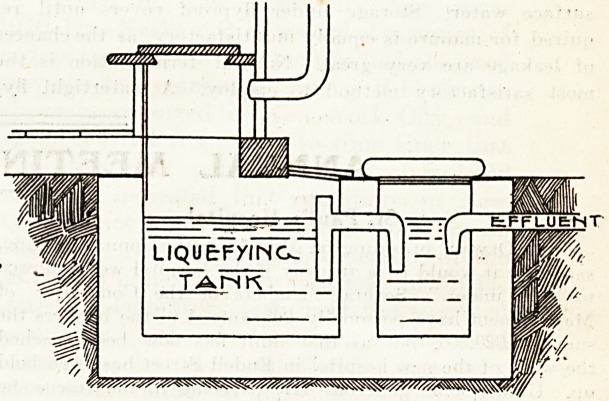# The Best Methods for Newly Claimed Areas

**Published:** 1921-05-28

**Authors:** 


### EXTEMPORISED SANITATION.

### The Best Methods for Newly Claimed Areas.

In the present outcry for cheap houses, and immense numbers of them, one fact seems to be almost entirely neglected, and that is the very important one of sanitation. Those who have spent all their lives in civilised towns are apt to forget that such things as water supplies, surface drainage, and general conservancy need very complicated organisations, much permanent material, and a large staff. Those who have lived in small country villages or in tropical countries, or any who have been with our Armies in the field, will have realised that a large number of men are employed and much time is spent, and that very uneconomically, in doing the work of sanitation under more primitive conditions.

Life shorn of its modern complications, the simple life, in fact, is one long struggle to get the raw material of life, and to dispose of the end products.

The first matter to consider in any housing scheme is the site on which the houses are to be built. If an extension of an existing township is under consideration, especially a large town, it often happens that the land most easily acquired, and at the cheapest rate, is that which private enterprise has for years for some reason or other avoided. For example, unless an expensive drainage system for surface and subsoil water is constructed, flat and low-lying localities are useless. A well-marked fall in the land is essential if a healthy colony is to be founded at small expense.

When a new town or village is to be built there is usually a freer choice for the site to be selected, but the same holds good that unless there is a natural slope to assist the surface drainage a large outlay will have to be sunk in the ground before it is fit to support a house.

No matter how suitable the site, some grading will have to be done, and a certain number of drains, either of the nature of ditches and gutters, or of underground sewers, will have to be built.

The roads may well serve as the main means of surface drainage; all that is needed is a sufficient number of roads running up from the low ground, along the sides of which run deep ditches or concrete gutters into which run the gutters of the roads which run along the slope of the hill. If well kept, deep ditches are the best; as they drain some of the subsoil water as well as the surface water. Roads are almost as important as drains, but may be made of any suitable local material, as at first they are not likely to have very heavy wear.

### Water Supply.

As regards water, if a local piped supply is available the erection of standard taps at suitable intervals is, where speed is necessary, a very good way of dealing with the subject. Later the main can have pipes to the various houses fixed as required. In really rural neighbourhoods it may be found that some natural source of water supply must be relied on. These are of three classes—springs, deep wells, shallow wells.

Springs may be made very efficient sources of pure water, which can safely be used pending the installation of a piped water supply. First, the spring must be cleaned out, right down to the rock or water-bearing stratum if possible. A pipe should then be fixed which will tap the water and lead it out some feet clear of the ground, so that buckets or other receptacles may be placed under the spout of the pipe. An alternative method is to build an iron or concrete tank and collect in it the water flowing from the spring. But on no account should receptacles be dipped into the spring. To prevent surface water from running into a spring a ditch, with a bank on the inner side, should be dug round it, the ditch leading into the stream well below the spot at which the water is to be collected.

Deep wells, in which a drill is used, are usually quite efficiently protected from contamination by the lining, but care should be taken that it is tightly screwed at the joints, and that it is driven into, if not through, the first impervious layer below the soil. The top of the well should be raised above the ground, and the pump should stand on a watertight base.

Shallow wells are never safe, though in individual instances they may prove to be satisfactory. They must be lined with some watertight material for as great a depth as is possible. They should be fitted with a strong watertight cover on which the pump should stand, and the top should be at least one foot above the ground, and so graded that water spilled is carried well away from the neighbourhood of the well. For wells where a pump is for any reason unsuitable one of the devices by which the bucket is enclosed in the windlass box, and automatically emptied through a spout, should be employed.

### The Disposal of Excreta.

If a piped water supply is available, a water-carriage system may be employed, though this necessitates a heavy outlay on drains. Cesspits lose in a great measure their chief drawback when a piped water supply, and not wells, is source of drinking-water, and so may reasonably be employed, thereby saving a heavy outlay on sewers.

Where a water-carriage system is impossible there remains some form of sanitary privy, and this to be satisfactory must fulfil the following conditions.

There must be no leakage into the water supply, there must be no opportunity for fly-breeding, and there must be freedom from offensive odours. They should also be capable of thorough and easy cleansing. And when this is arranged for, there is still the matter of the disposal of the collected excreta. The type often found in country cottages, where, at the bottom of the garden, is a seat over an open pit in a dilapidated shed, is without doubt the very worst possible. In more modern buildings a bucket which can be emptied into a pit is used. This makes the latrine less foul, but is practically no advance on the open pit, for it is never flyproof, is seldom emptied regularly, and the pit into which the bucket is emptied is no more efficient than the open pit in deterring flies, and the contamination of the subsoil water is equally certain.

In a sanitary privy a bucket must be used which almost touches the seat, which should form the top of a flyproof cupboard with a definite ventilation outlet. A well-fitting top, so made as to fall-to by its own weight, should effectually close the seat, and a door at the back should allow the bucket to be withdrawn for emptying. The floor should be cemented, and the inside of the seat tarred or creosoted. An alternative plan is to build a cement vault under the seat, the vault being provided with a flyproof trap at the back through which the contents can be removed. In this type there is no breaking of cans or buckets, but there is the certainty of fouling the ground when emptying the vault. The vault is intended to be emptied daily.

Any such plan leaves the disposal of excreta undealt with, and this is the larger part of the difficulty. Burning, as carried out in the Army and in many cantonments in India, needs much fuel, and stable litter, that most often used, is not easily obtained and is of great value as manure, as also is the excreta. Digging the excreta into the ground needs to be well done, or flies breed; and even when best done there is certain contamination of the surface water. Storage under flyproof covers until required for manure is equally unsatisfactory, as the chances of leakage are very great. Natural fermentation is the most satisfactory method to employ. A watertight flyproof tank of cement, brick, or other material is used, and into this the excreta are emptied, water is added, and the whole allowed to ferment, the resulting fluid being either pumped out, as in a cesspit, or else an effluent pipe is fitted by which the liquid is run off when it reaches a high enough level. It may be run into a large container or direct into the top soil of the garden.

This cesspit may with advantage be combined with the privy as in the attached sketch, which is self-explanatory. No disinfectant must be used, as it would interfere with efficient fermentation.

This should never be lost sight of, that none of the extemporised methods of sewage disposal are comparable with a water-carriage system, which should invariably be employed for preference, and substituted for other systems as soon as possible.

Household refuse may be divided into two classes, that which can be burned, and that which cannot. That which can should be burned, either on a weekly bonfire or in a stove or incinerator. While drying it should be stored in a galvanised-iron dustbin. Ashes, old tins, and other such incombustible matter must be removed, preferably at public expense, and may be dumped on low-lying ground, which is in time by this means raised and prevented from becoming a breeding ground for mosquitoes. In country districts kitchen waste is often in great demand for pig food. This is a quite satisfactory method of disposal, provided the pig pail is not left too long between emptyings.

## Figures and Tables

**Figure f1:**